# scSCC: A swapped contrastive learning‐based clustering method for single‐cell gene expression data

**DOI:** 10.1002/qub2.85

**Published:** 2025-02-05

**Authors:** Xiang Wang, Sansheng Yang, Hongwei Li

**Affiliations:** ^1^ School of Mathematics and Physics China University of Geosciences Wuhan China; ^2^ Wuhan Maritime Communication Research Institute Wuhan China

**Keywords:** single‐cell RNA‐seq, clustering, contrastive learning, swapped prediction

## Abstract

Cell clustering plays a pivotal role in deciphering the intricacies of cell types, facilitating subsequent cell annotation endeavors within scRNA‐seq data analysis. In this paper, we propose a novel swapped contrastive clustering algorithm for scRNA‐seq data called scSCC. scSCC combines two contrastive learning modules, namely the instance contrastive learning module and the swapped prediction module, to extract clustering‐friendly cell representations. Through the combination of swapped prediction module and instance contrastive learning module, scSCC can retrieve disentangled cell representations and amplify the clustering signals in the latent space, leading to satisfactory clustering performance. Different from existing contrastive‐learning‐based scRNA‐seq data clustering algorithms, the swapped prediction module of scSCC injects clustering signals to the latent space through some clustering prototypes. The swapped prediction module encourages cells of the same cluster to gravitate toward the common clustering prototype and naturally stay away from other prototypes in the latent space, hence cell representations obtained by scSCC are more clustering‐friendly compared to other algorithms. Experimental results on real scRNA‐seq datasets show that scSCC achieves improved clustering performance compared with the benchmark methods. The ablation study on two contrastive modules exhibits the promotion by the combination of instance learning module and swapped prediction module. The source codes are available at the GitHub website (EnchantedJoy/scSCC).

## 1 INTRODUCTION

The rapid development of single‐cell RNA sequencing (scRNA‐seq) technology allows researchers to analyze gene expression at the resolution of individual cells, providing unprecedented opportunities for studying the regulatory relationships between genes, the heterogeneity among different cells, and the mechanisms of cell interactions [1, 2, 3]. In recent years, with the decrease in sequencing costs and the introduction of new protocols, many scRNA‐seq datasets have been established, which also provide huge opportunities and challenges for scRNA‐seq data analysis. Unsupervised clustering is one of the most essential approaches to identify cell populations, which plays a vital role in subsequent analysis, such as cell type annotation and marker gene identification. However, due to the phenomenon known as “the curse of dimensionality” [4], some classical clustering algorithms are challenging to implement on scRNA‐seq data. Effective clustering algorithms are in desperate demand.

scRNA‐seq data is characterized by high dimensionality, high sparsity, and strong noise. In response to these characteristics, new clustering algorithms have been proposed in recent years. These clustering algorithms typically follow a two‐stage process. They first apply dimensionality reduction algorithms, such as principal component analysis (PCA) [5], to reduce the dimensionality and deal with “the curse of dimensionality”. Then, a clustering algorithm, such as K‐means algorithm [6] or Louvain algorithm [7], will be adopted to separate cells to different clusters. For instance, Seurat [8] first finds numerous mutual nearest neighbor (MNN) pairs based on the distance metric between cells, and then applies the Louvain algorithm to cluster cells. CIDR [9] first imputes the gene expression matrix, and then utilizes the hierarchical clustering algorithm [10] to cluster the data processed by PCA. SIMLR [11] first utilizes some kernel functions to construct a robust similarity matrix between cells, and then uses the spectral clustering algorithm [12] to obtain clustering assignments based on the similarity matrix.

With the development of deep learning, autoencoder‐based models are widely applied to perform the dimensionality reduction process and some distribution‐based decoders are designed to fit the distribution of scRNA‐seq data. For instance, deep count autoencoder (DCA) [13] utilizes the zero‐inflated negative binomial (ZINB) distribution to model scRNA‐seq data, which helps the network to learn cell representations. scziDesk [14] adopts a pre‐training phase based on DCA, and then combines soft K‐means [15] algorithm and deep embedding clustering (DEC) [16] network to cluster cell representations. scAce [17] utilizes a ZINB variational autoencoder to obtain cell representations and adopts a cluster merging strategy to promote the clustering results. Meanwhile, with the development of graph neural network (GNN) [18, 19], GNN‐based algorithms are gradually applied to scRNA‐seq data. For example, graph‐sc [20] constructs a bipartite graph based on gene expression matrix to consider interactions between cells and genes, and then utilizes graph autoencoder model to learn cell representations.

As both autoencoder‐based models and graph‐based models are highly dependent on the hypothesis of data distribution, this hypothesis can also limit the effectiveness of the model to some extent. In light of this, some contrastive‐learning‐based algorithms have been proposed. Contrastive learning is known as a new instance‐based paradigm for unsupervised learning, and some contrastive‐learning‐based methods have been successfully applied in the field of computer vision, such as SimCLR [21], MoCo [22], and SwAV [23]. These algorithms can perform image clustering very well without the predefined hypothesis of data distribution, greatly narrowing the gap between unsupervised learning and supervised learning. Contrastive learning methods first obtain two different augmented forms of the same data through a series of pretext tasks which are reflected in computer vision field as cropping, flipping, blurring, etc. The pretext tasks generate two different visions of the same data point and increase the differences between different samples, hence we can perform contrastive learning between different visions and different samples. After projecting the augmented data onto the hypersphere space, the two matched augmented data of the same data point (e.g., positive samples) are pulled closer, while different pairs of augmented data (negative samples) are pushed apart under the contrastive constraint. In this contrastive manner, data points can be separated from each other in the latent space, leading to more representative embeddings of samples.

Recently, there have been some attempts to apply contrastive learning to scRNA‐seq data. For example, CLEAR [24] removes the batch effect by performing contrastive learning on cells of different batches based on MoCo network, which utilizes an offline queue to stack negative samples and slightly update parameters of the encoder in a momentum updating manner. contrastive‐sc [25] introduces random mask and noise addition to construct pretext tasks, and then obtains cell representations by optimizing InfoNCE [26] loss based on SimCLR architecture. scNAME [27] utilizes the offline memory bank to stack negative samples and combines contrastive learning and soft K‐means to refine cell representations. The reconstructions of gene expression matrix and the mask matrix are then merged into scNAME, and DEC clustering module is finally used to fine‐tuning the cell representations. scGPCL [28] uses a graph neural network to learn cell representations and combines a contrastive learning module to enhance cell representations. However, these contrastive clustering methods only consider the instance learning, without explicitly characterizing the clustering structure in the latent space during the contrastive training phase, hence the cell representations learned by these contrastive networks may not be suitable for clustering. Some contrastive learning‐based methods attempt to extract clustering information from the learned cell representations. For example, ScCCL [29] utilizes a classifier to obtain fake labels and performs contrastive learning on these fake labels to refine cell representations implicitly, leading to the enhancement of clustering signals within cell representations.

Inspired by these preceding endeavors in clustering analysis, we propose a novel swapped contrastive clustering method for scRNA‐seq data named scSCC to extract cell representations in a contrastive manner and utilize the swapped prediction module to amplify the clustering signals during the contrastive training phase. scSCC first obtains positive and negative sample pairs through data augmentation module, and then scSCC projects the augmented data onto a low‐dimensional hypersphere space to obtain cell representations, which are then refined through the combination of instance contrastive learning module and swapped prediction module. Different from existing single‐cell algorithms based on contrastive learning, scSCC focuses more on the extraction of the clustering structure of the low‐dimensional representations of cells. According to the swapped prediction module, cells of the same category get close to each other in a low‐dimensional space since they tend to be distributed around the same prototype, and at the same time they naturally generate boundaries from cells of different categories. Notably, distinct from ScCCL, our proposed scSCC explicitly enhances the clustering structure of cell representations with the help of the swapped prediction module. Therefore, the clustering signals are amplified in the low‐dimensional space and cell representations obtained by scSCC are naturally more clustering‐friendly. The cluster labels are finally acquired by applying K‐means to the ultimate cell representations. Compared with existing customized scRNA‐seq data clustering methods on several datasets, scSCC achieves excellent clustering performance on these selected datasets. Besides, according to the 2D *t*‐distributed stochastic neighbor embedding (*t*‐SNE) [30] visualization plots of cell representations, scSCC generates distinct margins between different categories, which further verifies the superior clustering structure of the embedding space.

## 2 RESULTS

This section presents the performance of scSCC on the benchmark datasets. We first introduce an overview of the proposed scSCC method, followed by the descriptions of comparison methods. Then, we demonstrate the clustering performance of scSCC and present visualizations of the well‐structured cell representations learned by scSCC. Additionally, an ablation study is conducted to demonstrate the individual impact of each module, and subsequently, the influence of the augmentation module is finally illustrated.

### 2.1 The overview of scSCC

scSCC is a new extension of contrastive learning on scRNA‐seq data, and it is a contrastive clustering method for scRNA‐seq data based on swapped prediction. Since scSCC is used to deal with scRNA‐seq data clustering task, we first give a description of scRNA‐seq clustering problem here:

For a given preprocessed gene expression matrix $X={\left(x_{1},x_{2},\text{⋯},x_{m}\right)}^{T},x_{i}\in R^{n},i=1,2,\text{⋯},m,$ where *m* is the number of cells, *n* is the number of selected highly variable genes, $x_{i}$ is the expression vector of cell *i*. We need to first find an embedding map $f:R^{n}\to R^{d},d\ll n,$ where *d* is the dimension of cell representations in the latent space. Then, as for the cell representations $Z=\left\{z_{i}|z_{i}=f\left(x_{i}\right),i=1,2,\text{⋯},m\right\},z_{i}$ is the representation of cell *i*, we need to obtain a separation as follows:

$$\begin{array}{l} T=\left\{C_{j}|j=1,2,\text{⋯},K\right\},\\ s.t.\;Z=C_{1}\cup C_{2}\cup \text{⋯}\cup C_{K},C_{i}\cap C_{j}=\emptyset ,i\neq j, \end{array}$$

where $C_{j}$ represents cluster *j*, the separation T is the ultimate clustering result.

**FIGURE 1:**
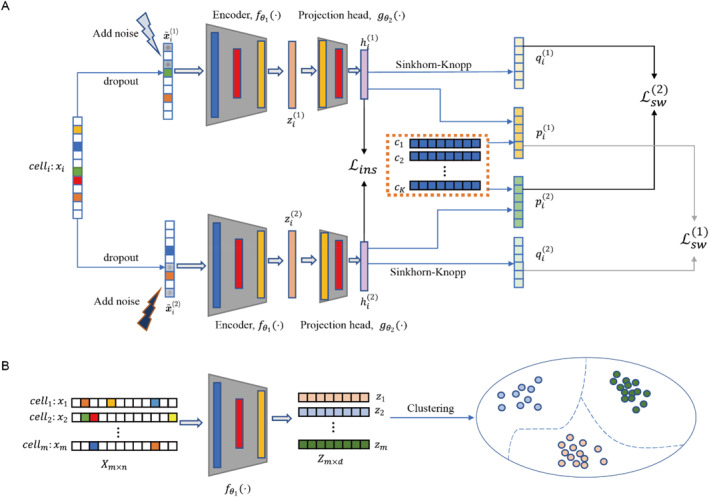
**The flowchart of scSCC**. (A) The network architecture of scSCC. For the preprocessed gene expression matrix, scSCC first obtains augmented visions of cells via the augmentation module, and then extracts cell representations through the combination of the instance contrastive learning module and the swapped prediction module. (B) The clustering phase. For the gene expression matrix, scSCC finally extracts cell representations by the encoder and utilizes the K‐means algorithm to perform the clustering phase.

scSCC’s primary objective is to extract well‐structured cell representations by employing a neural network architecture based on contrastive learning. The framework comprises three crucial modules: the data augmentation module, the instance contrastive learning module, and the swapped prediction module, as depicted in Figure 1. We here introduce these three modules as follows:

- ***Data augmentation module*.** To perform contrastive learning, a data augmentation module is utilized to generate two different visions of the given scRNA‐seq data. The input of this module is the preprocessed expression matrix $X={\left(x_{1},x_{2},\text{⋯},x_{m}\right)}^{T}\in R^{m\times n}.$ Through this augmentation module, we obtain two augmented visions ${\tilde{X}}^{\left(k\right)}={\left({\tilde{x}}_{1}^{\left(k\right)},{\tilde{x}}_{2}^{\left(k\right)},\text{⋯},{\tilde{x}}_{m}^{\left(k\right)}\right)}^{T}\in R^{m\times n}$ of the given expression matrix **X**, where *k* = 1,2 denotes different vision of expression matrix.
- ***Instance contrastive module*.** The instance contrastive learning module compresses cell expression features into a latent space to extract disentangled cell representations. This module consists of two parts: a feature extractor and a projection head. The feature extractor takes the augmented data ${\tilde{X}}^{\left(k\right)}$ as input and outputs good cell representation matrix ${\tilde{Z}}^{\left(k\right)}={\left({\tilde{z}}_{1}^{\left(k\right)},{\tilde{z}}_{2}^{\left(k\right)},\text{⋯},{\tilde{z}}_{m}^{\left(k\right)}\right)}^{T}\in R^{m\times d}\left(d\ll n\right),$ where *k* = 1,2 denotes different vision of augmentation and *d* denotes the dimension of the representation space. The representation matrix ${\tilde{Z}}^{\left(k\right)}$ is then projected to a deep feature space via the projection head, and we obtain the deep feature matrix ${\tilde{H}}^{\left(k\right)}={\left({\tilde{h}}_{1}^{\left(k\right)},{\tilde{h}}_{2}^{\left(k\right)},\text{⋯},{\tilde{h}}_{m}^{\left(k\right)}\right)}^{T}\in R^{m\times t},$ where *t* denotes the dimension of the deep feature space. InfoNCE loss between ${\tilde{H}}^{\left(1\right)}$ and ${\tilde{H}}^{\left(2\right)}$ is calculated to supervise the network training.
- ***Swapped prediction module*.** scSCC utilizes a swapped prediction module to amplify clustering signals in the latent space. Given the deep feature matrix ${\tilde{H}}^{\left(k\right)}$, this module outputs the cell type assignment matrix ${\tilde{P}}^{\left(k\right)}={\left({\tilde{p}}_{1}^{\left(k\right)},{\tilde{p}}_{2}^{\left(k\right)},\text{⋯},{\tilde{p}}_{m}^{\left(k\right)}\right)}^{T}\in R^{m\times K},$ where *K* denotes the preset number of clustering prototypes. To perform swapped prediction, this module generates an optimal matching probability ${\tilde{Q}}^{\left(k\right)}={\left({\tilde{q}}_{1}^{\left(k\right)},{\tilde{q}}_{2}^{\left(k\right)},\text{⋯},{\tilde{q}}_{m}^{\left(k\right)}\right)}^{T}\in R^{m\times K}$ for ${\tilde{P}}^{\left(k\right)}$. The cross entropy between ${\tilde{Q}}^{\left(k\right)}$ and ${\tilde{P}}^{\left(k\right)}$ is calculated to update the clustering prototypes and promote the clustering results.

scSCC takes the preprocessed expression matrix as input, and then generates augmented data through the data augmentation module. Subsequently, scSCC extracts clustering‐aware cell representations under the synergistic combination of instance contrastive learning module and swapped prediction module. Finally, a K‐means algorithm is performed to the cell representations to obtain the ultimate cell type assignments.

### 2.2 scSCC performs accurate clustering on scRNA‐seq data

In order to evaluate the clustering performance of scSCC, we compare the clustering results over 10 public datasets from different species and different organs (See more details in Section 4.6). These 10 datasets are respectively referred to as Muraro [31], Adam [32], Young [33], Baron [34], Macosko [35], Tosches [36], Bach [37], Trachea [38], Lung [38] and Spleen [38] (Table 1). To demonstrate the satisfactory clustering performance of scSCC, we compare scSCC with seven clustering algorithms developed for scRNA‐seq data based on the citation number and the network architecture, including Seurat [8], CIDR [9], SIMLR [21], scziDesk [14], graph‐sc [20], contrastive‐sc [25] and scNAME [27] (See more details in Section 4.6). To obtain a robust result, we set 10 random seeds and adopt the median value of 10 seeds as the ultimate result for each deep learning‐based benchmark method. For all the benchmark methods, we adopt the proposed default parameter settings of specific benchmark method for a fair comparison. We utilize adjusted Rand index (ARI) [39] and normalized mutual information (NMI) [40] to benchmark the clustering performance, which are widely used for evaluation [14, 17, 27, 41]. Meanwhile, we employ silhouette score (SC) [42] and Davies‐Bouldin index (DBi) [43] to provide a more comprehensive assessment of the clustering results.

**TABLE 1:** Details of datasets.

Dataset	Platform	Size (cells × genes)	Cell types
Muraro	CEL‐seq2	2122 × 19,046	9
Adam	Drop‐seq	3660 × 23,797	8
Young	10× Genomics	5685 × 33,658	11
Baron	inDrop	8569 × 20,125	14
Macosko	Drop‐seq	14,653 × 11,422	39
Tosches	Drop‐seq	18,664 × 23,500	15
Bach	10× Genomics	23,184 × 19,965	8
Trachea	Smart‐seq2	1350 × 23,341	4
Lung	Smart‐seq2	1676 × 23,341	11
Spleen	10× Genomics	9552 × 23,341	5

**FIGURE 2:**
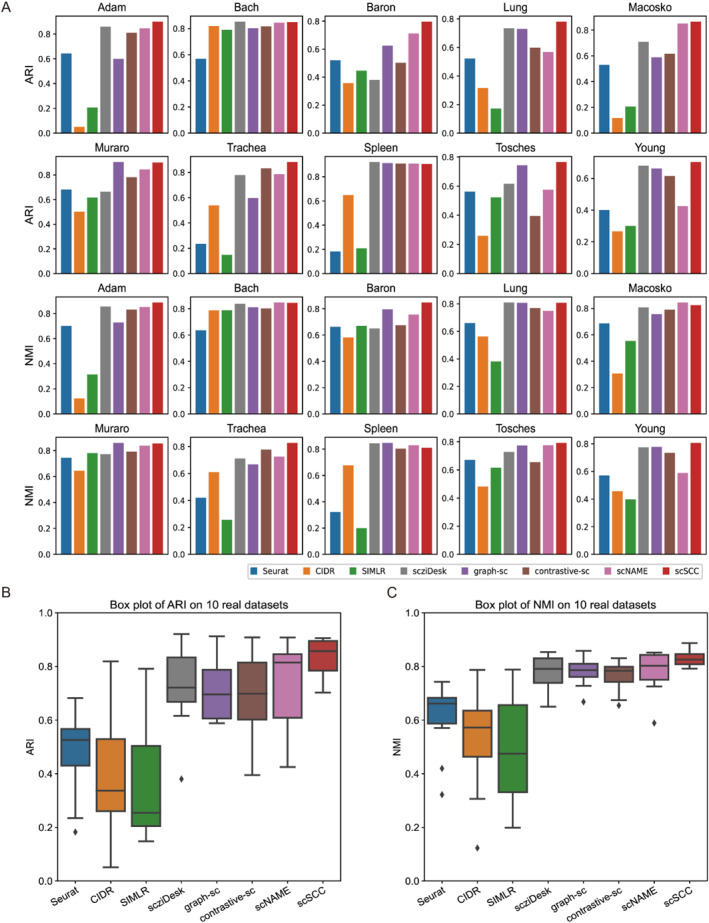
**Comparison results of ARI and NMI**. (A) Bar charts of ARI and NMI scores of the eight algorithms on 10 datasets. The first two rows are ARI scores and the last two rows are NMI scores. (B) Boxplot of ARI scores of the eight algorithms over 10 datasets. (C) Boxplot of NMI scores of the eight algorithms over 10 datasets. In the boxplot, the center line, box limits and whiskers denote the median, upper and lower quartiles, and 1.5 × interquartile range, respectively.

We first compare the performance of eight methods on each specific dataset, and the ARI and NMI scores are shown in Figure 2A (Tables S1 and S2). The first two rows represent the ARI scores across 10 benchmark datasets, and the following two rows represent the NMI scores across 10 benchmark datasets. We can see that scSCC outperforms other methods on seven datasets with highest ARI scores and ranks second on Bach and Muraro datasets without significant differences from the first place. Meanwhile, scSCC also outperforms other methods on 7 of these 10 datasets and still ranks top 3 on the remaining 3 datasets according to the NMI scores. Notably, we can see that on some datasets, such as Baron and Trachea datasets, scSCC can achieve more satisfactory performance and outperform other benchmark methods with significant improvements. Additionally, the SC scores for scSCC are the highest and the DBi scores for scSCC are the lowest on 9 datasets (Figure S1), demonstrating the superior clustering performance of scSCC.

For purpose of comparing the comprehensive performance, we evaluate the performance of each benchmark method across 10 datasets and present the boxplots of ARI scores in Figure 2B and NMI scores in Figure 2C. According to the boxplots, we can intuitively see that the box of scSCC is the narrowest but has the highest median score, which shows that scSCC achieves best performance across all datasets based on both ARI and NMI scores. Hence, we can see that the cell representations extracted by scSCC are more clustering‐friendly. In addition, the boxplots simultaneously illustrate the robustness of scSCC, as scSCC exhibited the minimum variance across 10 benchmark datasets.

Moreover, according to the selection of benchmark datasets, we can see the number of cell types differs from 4 to 39. However, scSCC can accurately cluster these datasets with various numbers of cell types and carry out a robust performance, implicating its potential for extensive applications. Furthermore, to rigorously evaluate the clustering performance of scSCC, we conducted comparisons with two more recent clustering algorithms (scAce [17] and scGPCL [28]). The results (Figure S2, Tables S3 and S4), based on ARI and NMI metrics, consistently demonstrated that scSCC exhibited the best performance on the benchmark datasets.

To demonstrate the capacity of scSCC, we measured the running time of scSCC and other four deep learning‐based methods under the condition of sufficient computing power (Figure S3). scSCC ranked third in terms of running time, being slightly slower than contrastive‐sc and graph‐sc, but faster than scNAME and scziDesk. As for the GPU memory usage, scSCC maintained stable memory consumption across each dataset since we trained scSCC using a mini‐batch training approach.

### 2.3 scSCC extracts clustering‐friendly cell representations

**FIGURE 3:**
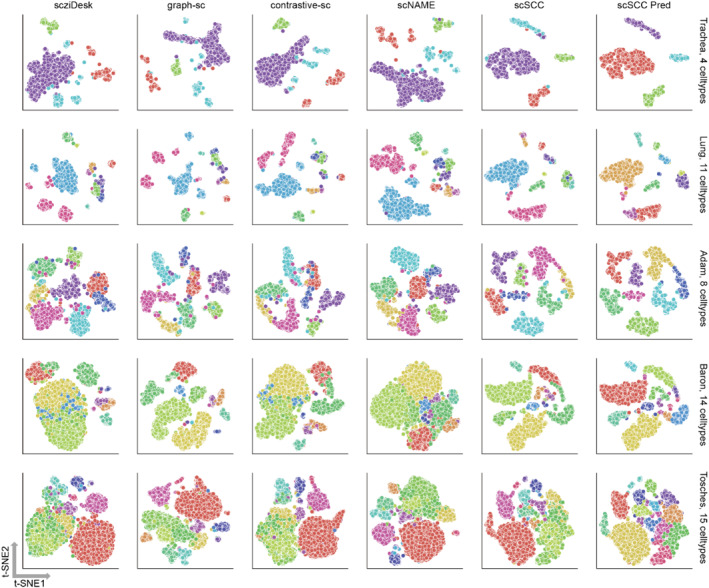
***t*‐SNE visualization of cell representations**. *t*‐SNE visualization of scziDesk, graph‐sc, scNAME, contrastive‐sc and scSCC on five datasets. Each point denotes a cell and different colors represent different true labels. “scSCC Pred” refers to results visualized with predicted labels.

In order to intuitively compare the clustering performance and analyze the cell representations extracted by scSCC, we select five datasets and visualize the embedding spaces of scSCC and other four deep learning‐based benchmark methods by projecting the cell representations into two dimensions using *t*‐SNE [30]. Visualization results are shown in Figure 3, and the full visualization results of scSCC on all 10 datasets are shown in the Supplementary Figure S4 and S5 in Supporting Information S1. Each row in Figure 3 exhibits the *t*‐SNE visualization results of different benchmark methods on the same dataset. In each subplot, each point refers to a cell and cells are colored by cell types according to the ground‐truth labels.

As shown in Figure 3, scSCC can obtain cell representations with distinct boundaries between different clusters, which further clarifies cell representations extracted by scSCC are clustering‐friendly. Moreover, clustering results of the cell representations extracted by scSCC are more consistent with the cell types, while other benchmark methods usually mix different cell types in the embedding space.

Notably, for the Trachea dataset, only scSCC obtains four pure clusters and forms clear margins between different clusters. For all other four methods, cells of the same cell type are separated apart. For the Baron dataset, only scSCC separates cells of different types apart, while other methods just mix cells with different cell types, hence cell representations extracted by scSCC are equipped with better clustering structure and are more clustering‐friendly.

Furthermore, to demonstrate the enhancement of clustering signals in the embedding space, we employ the K‐means algorithm on the input of scSCC for each dataset. The results indicated that cell representations extracted by scSCC lead to an enhancement of clustering performance, achieving higher ARI and NMI scores across 9 datasets (Figure S6 and S7).

### 2.4 Swapped prediction module enhances the performance of scSCC

As we mentioned that the combination of the instance contrastive learning module and the swapped prediction module is one of our innovations. In order to further evaluate the effectiveness of the swapped prediction module, we conduct an ablation study to demonstrate the effectiveness of combining the swapped prediction module and the instance contrastive learning module.

**FIGURE 4:**
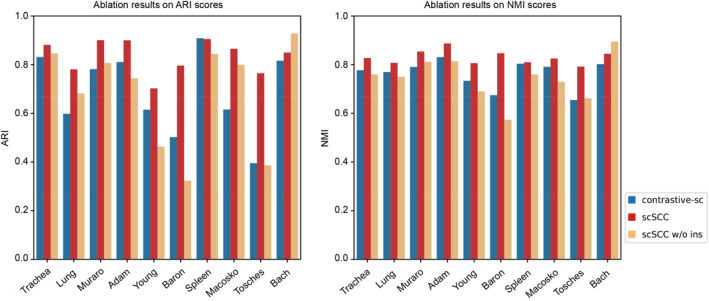
**Ablation results of ARI and NMI**. Shapes of datasets grow from left to right. Here “scSCC w/o ins” denotes the ablated version without instance module and “contrastive‐sc” represents the ablated version without swapped prediction module.

In this study, we initiate the process by excluding the instance contrastive module from the scSCC framework, resulting in the creation of a modified scSCC version which we refer to as “scSCC w/o ins”. This ablated version is used to verify the effectiveness of the combination of two contrastive modules. Subsequently, we proceed to eliminate the swapped prediction module from scSCC, leading to the development of the second ablated version. This ablated version is used to exhibit the promotion brought by the swapped prediction module. Since the second ablated version of scSCC is somewhat similar to contrastive‐sc, we directly use contrastive‐sc to replace the second ablated version. All the three versions are compared on the 10 benchmark datasets and the ARI scores and NMI scores are compared in Figure 4.

We observed that upon removing each contrastive module, respectively, the scores exhibited varying degrees of decline on most of these datasets, which validates the superiority of combining these two modules. Moreover, comparing the *t*‐SNE visualizations of contrastive‐sc and scSCC (shown in Figure 3), we can clearly see that scSCC presents a better clustering structure, which exhibits that the swapped prediction module can amplify clustering signals and scSCC can extract clustering‐friendly cell representations.

The effectiveness of the swapped prediction module can be intuitively explained as follows: cells of the same cell type tend to be distributed around the same prototype under the guidance of the swapped prediction module, while cells with different labels will simultaneously be distributed around distinct prototypes. This process ultimately leads to an enhancement to the instance contrastive learning module and the generation of more clustering‐friendly cell representations.

### 2.5 Influence of data augmentation module

We here explore the influence of the data augmentation module, as the data augmentation step plays an important role in contrastive learning‐based neural networks [21, 23, 25, 27].

In the workflow of scSCC, scSCC utilizes random mask and random noise to generate the data augmentation module. We change the noise weight α from 0.01 to 1 (Figure S8), and the results indicate that a slight noise ratio can achieve higher ARI and NMI scores. Since the masking probability is high and the noise proportion is very slight, we here pay more attention to the influence of the masking probability $p_{m}$ and display the performance of scSCC under different settings of $p_{m}$.

**FIGURE 5:**
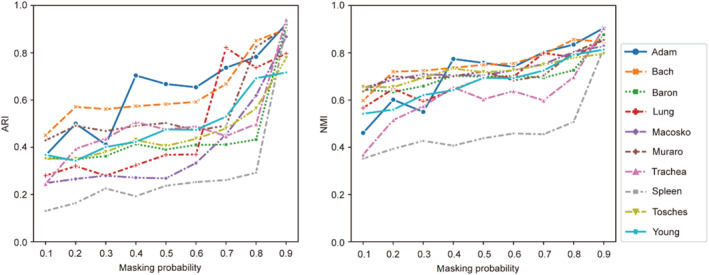
**Influence of masking probability** $p_{m}$. We show the influence of the masking probability $p_{m}$ and $p_{m}$ grows from 0.1 to 0.9 in steps of 0.1. The results of different datasets are decorated with distinct colors and markers.

We make $p_{m}$ gradually increase from 0.1 to 0.9 in intervals of 0.1. The line plots of ARI and NMI are shown in Figure 5. We can see that $p_{m}$ has a very large impact on the clustering performance and a higher masking probability setting usually obtains a better clustering result. This is corresponding to the characteristics of scRNA‐seq data since scRNA‐seq data is characterized by high sparsity and strong noise. High masking probability seems to only keep the essential information in the gene expression matrix and introduces more differences between different cells, which assists the encoder to separate cells from each other in the contrastive training phase.

To further explore the effectiveness of the masking strategy, we calculate the cell similarity under different masking probabilities using the preprocessed expression vectors and PCA expression vectors, respectively (Figure S9). We observed that a high masking probability can decrease the similarity of the input of scSCC but the similarity still remains in the PCA space. Moreover, a high masking probability can make the similarity between cells of the same type more consistent, which further prevents cells of the same type from being divided into different subclusters and protects the embedding space (Figures S10 and S11). We attribute this to the capability of the data augmentation module to force the encoder to preserve more essential information of gene levels, hence the cell representations learned by scSCC are more consistent with cell types.

## 3 DISCUSSION AND CONCLUSION

Cell clustering is a fundamental challenge to scRNA‐seq data analysis and is crucial for downstream tasks such as cell annotation and marker gene identification. In this paper, we propose a novel swapped prediction‐based clustering algorithm for scRNA‐seq data called scSCC. Compared with existing deep learning‐based clustering algorithms for scRNA‐seq data, scSCC learns cell embeddings in an instance‐based contrastive manner and does not depend on the predefined distribution of scRNA‐seq data. While compared with other contrastive clustering algorithms for scRNA‐seq data, scSCC facilitates clustering structure of the embedding space via the combination of swapped prediction and instance contrastive learning, hence cell representations extracted by scSCC can be more clustering‐friendly.

We show scSCC achieves satisfactory performance compared with other seven customized scRNA‐seq data clustering algorithms over 10 public datasets. The visualization results exhibit the clustering‐friendly structure of cell representations extracted by scSCC, which is reflected by the distinct boundaries between categories in the 2D *t*‐SNE visualization space. As a contrastive learning‐based algorithm, we also discussed the influence of the augmentation module on the clustering performance and a high masking probability seems to generate more discriminative negative samples according to the results.

In a general sense, scSCC is a novel dimensionality reduction method via a swapped prediction strategy, which has never been used in scRNA‐seq data analysis to the best of our knowledge. Notably, since scSCC is a pure instance‐driven clustering method, it may be extended to multi‐omics data with the help of a more suitable augmentation module and this will be our further work.

As more and more scRNA‐seq data becomes available, we believe that scSCC will serve as a valuable tool in executing the pivotal clustering step in scRNA‐seq data analysis.

## 4 MATERIALS AND METHODS

This section describes the algorithmic principles of scSCC, including data preprocessing and model architecture, providing a theoretical foundation for the scSCC method. Moreover, the details of the benchmark methods, benchmark datasets, and the evaluation metrics are also illustrated in this section.

### 4.1 Data preprocessing

scSCC follows the regular preprocessing steps for scRNA‐seq data and these are implemented in python package Scanpy [44]. For the given raw expression matrix, the genes expressed in less than two cells and the cells with no gene expression are both discarded. We denote the filtered matrix as $Y=\left(y_{ij}\right)\in R^{m\times n_{0}},m,n_{0}$ represent cell number and gene number, respectively. Next, this expression matrix **Y** is normalized by the library size factor $s\in R^{m}$ which represents the total expression number of each cell, and the natural logarithm is used to improve the robustness of network training. Hence we get the preprocessed expression matrix $\hat{X}=\left({\hat{x}}_{ij}\right)\in R^{m\times n_{0}},$

(1) $$\left\{\begin{array}{l} {\hat{x}}_{ij}=\log\left(\frac{s_{mid}}{s_{i}}\cdot y_{ij}+1\right),\\ s={\left(s_{1},s_{2},\text{⋯},s_{m}\right)}^{T}\in R^{m},\\ s_{i}=\sum_{j=1}^{n_{0}}y_{ij},s_{mid}=\text{median}\left(s\right), \end{array}\right.$$

where $s_{mid}$ denotes the median of s.

We then select top *n* highly variable genes according to their dispersion rankings as proposed in scziDesk [14] and then apply z‐score normalization to each gene, such that the expression level of every gene has zero mean and unit variance across cells. We denote the ultimate preprocessed expression matrix as $X=\left(x_{ij}\right)\in R^{m\times n},$ *n* denotes the number of highly variable genes and is defaulted to be 2,000 in scSCC.

### 4.2 Data augmentation

Data augmentation is also known as pretext task in contrastive learning. As we mentioned before, the data augmentation module is used to introduce differences between positive samples and negative samples. Considering the high sparsity and high noise in scRNA‐seq data, we adopt a random masking strategy to mask the noise information and generate more discriminative negative samples to help the network extract disentangled representations. Meanwhile, to enhance the robustness of network training, we add slight  Gaussian noise to the masking expression vector and then feed the augmented data to the neural network. The combination of these two strategies generates our data augmentation module. Specifically, for the given gene expression vector $x_{i}$ of cell *i*, we randomly mask $x_{i}$ twice and add Gaussian noise to obtain the augmented pair $\left\{{\tilde{x}}_{i}^{\left(1\right)},{\tilde{x}}_{i}^{\left(2\right)}\right\},$ that is:

(2) $$\left\{\begin{array}{l} {\tilde{x}}_{i}^{\left(1\right)}=m_{1}⊙x_{i}+\alpha \cdot \varepsilon_{1},\\ {\tilde{x}}_{i}^{\left(2\right)}=m_{2}⊙x_{i}+\alpha \cdot \varepsilon_{2}, \end{array}\right.$$

where $m_{k}={\left(m_{1}^{k},m_{2}^{k},\text{⋯},m_{n}^{k}\right)}^{T}\in {\left\{0,1\right\}}^{n}$ is the mask vector, and $m_{i}^{k}∼B\left(1-p_{m}\right)$. Here we use $B\left(1-p_{m}\right)$ to denote Bernoulli distribution with probability $1-p_{m}$. $\varepsilon_{k}={\left(\varepsilon_{1}^{k},\varepsilon_{2}^{k},\text{⋯},\varepsilon_{n}^{k}\right)}^{T}\in R^{n}$ is the Gaussian noise vector, and each $\varepsilon_{i}^{k}∼N\left(0,1\right)$, and ⊙ refers to Hadamard product. α is a hyperparameter to control the proportion of noise.

For *N* samples ${\left\{x_{i}\right\}}_{i=1}^{N}$ in the same minibatch, we can obtain *N* pairs of augmented data ${\left\{{\tilde{x}}_{i}^{\left(1\right)},{\tilde{x}}_{i}^{\left(2\right)}\right\}}_{i=1}^{N}$ via this data augmentation layer,

(3) $${\tilde{x}}_{i}^{\left(k\right)}=C\left(x_{i}\right),k=1,2,i=1,2,\text{⋯},N,$$

where C(⋅) denotes this data augmentation layer. ${\tilde{x}}_{i}^{\left(1\right)}$ and ${\tilde{x}}_{i}^{\left(2\right)}$ are regarded as positive pair samples for a fixed cell *i*, and the remaining 2N−2 samples are regarded as negative samples of ${\tilde{x}}_{i}^{\left(1\right)}$ and ${\tilde{x}}_{i}^{\left(2\right)}$.

### 4.3 Instance contrastive learning

Since the cell expression features in the raw space are filled with noise, we first utilize the instance contrastive learning module to extract disentangled cell representations in the latent space. The representation extractor is an encoder consisting of three layers of fully connected neural networks with ReLU activation unit. We use *E* to denote the encoder and use $f_{\theta_{1}}\left(\cdot \right)$ to denote the nonlinear map fitted by *E*. Similar to SimCLR [21], we concatenate a projection head *P* after *E* to reduce the information loss in the representation space during the training phase. The projection head *P* consists of two layers of fully connected neural networks with ReLU activation unit and we use $g_{\theta_{2}}\left(\cdot \right)$ to denote the nonlinear map fitted by *P*. Here $\theta_{1}$ and $\theta_{2}$ represent network parameters of *E* and *P* respectively. For the given *N* pairs of augmented cell expression vectors ${\left\{{\tilde{x}}_{i}^{\left(1\right)},{\tilde{x}}_{i}^{\left(2\right)}\right\}}_{i=1}^{N}$, we feed these data to *E* and *P* to extract cell representations,

(4) $$z_{i}^{\left(k\right)}=f_{\theta_{1}}\left({\tilde{x}}_{i}^{\left(k\right)}\right),h_{i}^{\left(k\right)}=g_{\theta_{2}}\left(z_{i}^{\left(k\right)}\right),i=1,2,\text{⋯},N,k=1,2,$$

where $z_{i}^{\left(k\right)}\in R^{d}$ denotes the cell representation of cell *i* under the augmentation vision *k*, and *d* is the dimension of latent representation space. $h_{i}^{\left(k\right)}\in R^{t}$ denotes the deep feature of cell *i* under the augmented vision *k*, and *t* is the dimension of the deep feature space. We perform contrastive learning on ${\left\{{\tilde{h}}_{i}^{\left(1\right)},{\tilde{h}}_{i}^{\left(2\right)}\right\}}_{i=1}^{N}$ to protect the structure of the representation space.

We apply InfoNCE [26] loss to accomplish the network training phase,

(5) $$L_{ins}=\frac{1}{2N}\sum_{i=1}^{N}\left(l_{i}^{\left(1\right)}+l_{i}^{\left(2\right)}\right),$$

(6) $$l_{i}^{\left(a\right)}=-\log\frac{\exp\left(\rho \left(h_{i}^{\left(1\right)},h_{i}^{\left(2\right)}\right)/\tau_{i}\right)}{\sum_{j=1}^{N}\sum_{k=1}^{2}\left[\exp\left(\rho \left(h_{i}^{\left(a\right)},h_{j}^{\left(k\right)}\right)/\tau_{i}\right)\right]},a=1,2,$$

(7) $$\rho \left(h_{i}^{\left(1\right)},h_{i}^{\left(2\right)}\right)=\frac{{\left(h_{i}^{\left(1\right)}\right)}^{T}\left(h_{i}^{\left(2\right)}\right)}{\left\|h_{i}^{\left(1\right)}\right\|\cdot \left\|h_{i}^{\left(2\right)}\right\|},$$

where $\tau_{i}$ is the temperature parameter used to smooth the similarity between samples. ρ(⋅,⋅) is a distance metric and we use cosine similarity here. The network parameters $\theta_{1},\theta_{2}$ are then optimized via back propagation.

### 4.4 Swapped prediction

As previously mentioned, the instance contrastive learning module can make cell representations be distributed uniformly in the latent space. However, the cell representations extracted by this module are not equipped with clustering structure. To enhance the clustering signals within the latent space, we introduce the swapped prediction module. By integrating this module with the instance contrastive learning module, we can develop clustering‐friendly cell representations that are well‐suited for clustering.

The swapped prediction module predicts the soft assignments of cells with the *K* clustering prototypes to inject clustering signals into the latent space. This module enforces the constraint that two different visions of the same cell should be equipped with the same probabilistic assignments, which is also consistent with our intuition. In this swapped prediction manner, cells with similar representations will get close to the same clustering prototype and far away from other prototypes; hence, cell representations will be more clustering‐friendly.

Same as instance contrastive learning, the swapped prediction step is also conducted on ${\left\{{\tilde{h}}_{i}^{\left(1\right)},{\tilde{h}}_{i}^{\left(2\right)}\right\}}_{i=1}^{N}$. In order to obtain cell type assignments, we initialize *K* clustering prototypes:

(8) $$C={\left(c_{1},c_{2},\text{⋯},c_{K}\right)}^{T},c_{j}\in R^{t},j=1,2,\text{⋯},K,$$

where $c_{j}$ is the *j*‐th clustering prototype, characterized as a vector of network parameters. *t* is the dimension of the projected feature space.

We can then obtain cell type assignment $p_{i}^{\left(k\right)}$ calculated by prototypes **C** and the deep feature $h_{i}^{\left(k\right)}$. For the paired positive samples, the assignments are:

(9) $$p_{i}^{\left(1\right)}=\text{softmax}\left(\frac{C\cdot g_{\theta_{2}}\left(f_{\theta_{1}}\left({\tilde{x}}_{i}^{\left(1\right)}\right)\right)}{\tau_{s}}\right),$$

(10) $$p_{i}^{\left(2\right)}=\text{softmax}\left(\frac{C\cdot g_{\theta_{2}}\left(f_{\theta_{1}}\left({\tilde{x}}_{i}^{\left(2\right)}\right)\right)}{\tau_{s}}\right),$$

where $\tau_{s}$ is the temperature parameter used to smooth the soft labels. The softmax function is used to output label probabilities.

Notably, in order to prevent model collapse during the training phase, we do not use label pairs ${\left\{{\tilde{p}}_{i}^{\left(1\right)},{\tilde{p}}_{i}^{\left(2\right)}\right\}}_{i=1}^{N}$ to conduct swapped prediction. Instead, we utilize the Sinkhorn–Knopp algorithm [45] to process the label pairs ${\left\{{\tilde{p}}_{i}^{\left(1\right)},{\tilde{p}}_{i}^{\left(2\right)}\right\}}_{i=1}^{N}$ and obtain the optimal matching probabilities denoted as ${\left\{{\tilde{q}}_{i}^{\left(1\right)},{\tilde{q}}_{i}^{\left(2\right)}\right\}}_{i=1}^{N}$. These optimal matching probabilities are then integrated into the training process [23] in a swapped prediction manner with ${\left\{{\tilde{p}}_{i}^{\left(1\right)},{\tilde{p}}_{i}^{\left(2\right)}\right\}}_{i=1}^{N}$. Hence, we obtain the swapped prediction loss:

(11) $$L_{sw}=-\sum_{i=1}^{N}\sum_{j=1}^{K}\left(q_{ij}^{\left(1\right)}\;\log\;p_{ij}^{\left(2\right)}+q_{ij}^{\left(2\right)}\;\log\;p_{ij}^{\left(1\right)}\right).$$

The network parameters $\theta_{1},\theta_{2}$ and **C** are then updated through back propagation.

### 4.5 scSCC

We finally elaborate the entire workflow of scSCC. For the given preprocessed expression matrix **X**,

(12) $$X={\left(x_{1},x_{2},\text{⋯},x_{m}\right)}^{T},x_{i}\in R^{n},i=1,2,\text{⋯},m.$$

We then feed **X** to the neural network to obtain cell representations and deep features, and the whole forward propagation process is summarized as follows:

(13) $$\left\{\begin{array}{l} {\tilde{X}}^{\left(k\right)}=C^{\left(k\right)}\left(X\right),\\ Z^{\left(k\right)}=f_{\theta_{1}}\left({\tilde{X}}^{\left(k\right)}\right),\\ H^{\left(k\right)}=g_{\theta_{2}}\left(Z^{\left(k\right)}\right),\\ P^{\left(k\right)}=\text{softmax}\left(H^{\left(k\right)}C^{T}/\tau_{s}\right),\\ Q^{\left(k\right)}=\text{Sinkhorn}\left(H^{\left(k\right)}C^{T}\right), \end{array}\right.$$

where *k* = 1,2 denotes different vision of augmented data and Sinkhorn(⋅) denotes the Sinkhorn algorithm.

We can then obtain the swapped prediction loss $L_{sw}$ and the InfoNCE loss $L_{ins}$ through swapped prediction module and instance contrastive learning module, respectively. By combining the two aforementioned losses, we arrive at the overall network loss,

(14) $$L=L_{sw}+\kappa L_{ins},$$

where κ is a hyperparameter to make a trade‐off between $L_{sw}$ and $L_{ins}$. We set κ=0.01 by default and change κ=1 when the number of cells exceeds 10,000, and the discussion of κ is shown in Tables S5 and S6. The temperature parameters of $L_{sw}$ and $L_{ins}$ are discussed in Figure S12, and we set $\tau_{i}=\tau_{s}=0.1$ as default based on the overall performance across 10 datasets. The default setting of the noise ratio was α=0.1. As discussed in Figure S8, we recommend to select α in [0.01,0.1]. The network parameters $\theta_{1},\theta_{2}$ and **C** are ultimately updated via back propagation, and the whole algorithm is illustrated in Algorithm 1.

During the training phase, we first apply Equation (14) to conduct a pretraining phase, and then utilize the swapped prediction loss $L_{sw}$ to refine the representations and the prototypes, which can be intuitively understood as we first extract disentangled representations with clustering information amplified and then refine the clustering structure to help the prototypes to distinguish hard negative samples. In order to fully leverage the labels $\tilde{S}$ predicted by prototypes, we employ the K‐means algorithm to obtain pseudo labels *S* and combine $\tilde{S}$ and *S* to make an early stopping for the neural network. When the ARI value between $\tilde{S}$ and *S* is larger than the preset threshold *tol*, the training phase meets the early stopping condition. After the network has been trained, we employ the K‐means algorithm to the cell representations $Z\in R^{m\times d}$ obtained by the neural network and consequently obtain the cell type assignment array $S\in {\left\{1,2,\text{⋯},K\right\}}^{m}$.

Algorithm **1. The algorithm of scSCC**

  **Input:** Raw expression matrix $X_{raw}$; number of cell types *K*; temperature parameters $\tau_{s},\tau_{i}$; mask probability $p_{m}$; noise weight α; weight of InfoNCE loss κ; batch size *N*; pretraining epochs $n_{pre}$; fine‐tuning epochs $n_{e}$; initialized parameters $\theta_{1},\theta_{2}$; initialized prototypes **C**; threshold *tol.*
  **Output:** cell embedding matrix Z; cell type assignment S.
1:  preprocess $X_{raw}$ to **X**, and initialize 
    ${\text{ARI}}_{\max}=-1$
2:  **for** epoch = 1 to $n_{e}$ **do**
3:    **for** $x_{i}\in \text{batch}$ **do**
4:     ${\tilde{x}}_{i}^{\left(1\right)}=m_{1}⊙x_{i}+\alpha \cdot \varepsilon_{1}$
5:     ${\tilde{x}}_{i}^{\left(2\right)}=m_{2}⊙x_{i}+\alpha \cdot \varepsilon_{2}$
6:     calculate embeddings and cell type 
       assignments by
7:     $z_{i}^{\left(1\right)}=f_{\theta_{1}}\left({\tilde{x}}_{i}^{\left(1\right)}\right),z_{i}^{\left(2\right)}=f_{\theta_{1}}\left({\tilde{x}}_{i}^{\left(2\right)}\right)$
8:     $h_{i}^{\left(1\right)}=g_{\theta_{2}}\left(z_{i}^{\left(1\right)}\right),h_{i}^{\left(2\right)}=g_{\theta_{2}}\left(z_{i}^{\left(2\right)}\right)$
9:     $q_{i}^{\left(1\right)}=\text{sinkhorn}\left(C\cdot h_{i}^{\left(1\right)}\right),p_{i}^{\left(1\right)}=\text{softmax}\left(C\cdot h_{i}^{\left(1\right)}/\tau_{s}\right)$
10:     $q_{i}^{\left(2\right)}=\text{sinkhorn}\left(C\cdot h_{i}^{\left(2\right)}\right),p_{i}^{\left(2\right)}=\text{softmax}\left(C\cdot h_{i}^{\left(2\right)}/\tau_{s}\right)$
11:     calculate $L_{sw}$ by (11)
12:     **if** $\text{epoch}>n_{pre}$ **then**
13:      $L_{ins}=0$
14:     **else**
15:      calculate $L_{ins}$ by (5)
16:     end if
17:      calculate total loss by (14) and 
      update parameters $\theta_{1},\theta_{2}$ and **C**
18:     **if **$\text{epoch}>n_{pre}$ **then**
19:      calculate cell type assignments 
       *S* and $\tilde{S}$ by K‐means and prototypes 
       respectively
20:      calculate ${\text{ARI}}_{cur}$ between *S* and $\tilde{S}$
21:      update ${\text{ARI}}_{\max}=\max\left({\text{ARI}}_{\max},{\text{ARI}}_{cur}\right)$
       and check early stopping 
       condition
22:     **end if**
23:    **end for**
24:  **end for**
25:  obtain ultimate cell embedding matrix Z

### 4.6 Benchmark datasets and methods

We use 10 publicly available datasets with annotated cell type labels to test performance of scSCC and specific information is shown in Table 1. These 10 datasets include three human datasets, six mouse datasets, and one turtle dataset. For convenience, we utilize author’s name to refer to the specific dataset, and then these datasets are referred to as Muraro [31], Adam [32], Young [33], Baron [34], Macosko [35], Tosches [36], Bach [37], Trachea [38], Lung [38] and Spleen [38]. Here we use organ names to refer to the last three datasets since they were developed by the same author. These datasets come from different platforms and cell numbers vary from 1,350 to 27,499, and hence can provide a comprehensive reflection of the performance of scSCC. Notably, as for Macosko dataset, we use the expression data filtered by scDCC [46] to conduct the experiment. The annotation methods and the accessions of these datasets are described in Table S7.

We compare scSCC with seven clustering algorithms developed for scRNA‐seq data based on the citation number and the network architecture, including Seurat [8], CIDR [9], SIMLR [21], scziDesk [14], graph‐sc [20], contrastive‐sc [25], and scNAME [27]. Seurat, CIDR, and SIMLR are traditional methods, and here traditional methods refer to methods without neural networks. scziDesk is a ZINB autoencoder‐based clustering method and graph‐sc is a GNN‐based method; here, we utilize these two methods to provide a comprehensive comparison with our contrastive‐learning‐based method scSCC since they were implemented by different neural network architectures. In addition, we also compare scSCC with two other contrastive‐learning‐based clustering methods contrastive‐sc and scNAME to exhibit the satisfactory clustering performance of scSCC. Specifically, contrastive‐sc is a contrastive learning method motivated by SimCLR [21] and scNAME is a combination method of contrastive learning and ZINB autoencoder. All these benchmark methods are available in their original papers.

### 4.7 Evaluation metrics

We select ARI [39], NMI [40], SC [42], and DBi [43] to measure the clustering results. ARI and NMI are calculated using the predicted labels and the ground‐truth labels, as formulated in Equations (15) and (16).

(15) $$\text{ARI}=\frac{\left(\begin{array}{l} n\\ 2 \end{array}\right)\left(a+d\right)-\left[\left(a+b\right)\left(a+c\right)+\left(c+d\right)\left(b+d\right)\right]}{{\left(\begin{array}{l} n\\ 2 \end{array}\right)}^{2}-\left[\left(a+b\right)\left(a+c\right)+\left(c+d\right)\left(b+d\right)\right]},$$

(16) $$\text{NMI}=\frac{-2\sum_{p=1}^{C_{U}}\sum_{q=1}^{C_{V}}\left|U_{p}\cap V_{q}\right|\log\frac{n\left|U_{p}\cap V_{q}\right|}{\left|U_{p}\right|\cdot \left|V_{q}\right|}}{\sum_{p=1}^{C_{U}}\left|U_{p}\right|\log\frac{\left|U_{p}\right|}{n}+\sum_{q=1}^{C_{V}}\left|V_{q}\right|\log\frac{\left|V_{q}\right|}{n}},$$

where *a* is the number of sample pairs assigned to the same category in *U* and *V*, *b* is the number of sample pairs assigned to the same category in *U* but differ in *V*, *c* is the number of sample pairs assigned to the same category in *V* but differ in *U*, *d* is the number of sample pairs assigned to different categories in *V* and *U*, $U_{p}$ consists of samples assigned to category *p* in *U*, $V_{q}$ consists of samples assigned to category *q* in *V*, $\left|U_{p}\right|$ denotes the cardinality of $U_{p}$, and *n* is total number of samples.

Both metrics are widely used to evaluate category consistency between two assignment vectors *U* and *V*. ARI values in [−1,1], and higher score implies better clustering result. NMI values in [0,1], and score closer to one means a better result.

SC and DBi are calculated by cell representations and the predicted labels. For the cell representations **Z** and a separation $\left\{C_{1},C_{2},\text{⋯},C_{K}\right\}$ with *K* clusters, the SC and DBi are calculated by Equations (17) and (18), respectively.

(17) $$\text{SC}=\frac{1}{m}\sum_{I=1}^{K}\sum_{x_{i}\in C_{I}}\frac{\min_{J\neq I}\frac{1}{\left|C_{J}\right|}\sum_{x_{j}\in C_{J}}d\left(x_{i},x_{j}\right)-\frac{1}{|C_{I}|-1}\sum_{x_{j}\in C_{I}}d\left(x_{i},x_{j}\right)}{\max\left(\frac{1}{|C_{I}|-1}\sum_{x_{j}\in C_{I}}d\left(x_{i},x_{j}\right),\min_{J\neq I}\frac{1}{\left|C_{J}\right|}\sum_{x_{j}\in C_{J}}d\left(x_{i},x_{j}\right)\right)},$$

(18) $$\text{DBi}=\frac{1}{K}\sum_{I=1}^{K}\max_{J\neq I}\left(\frac{\frac{1}{\left|C_{I}\right|}\sum_{x_{i}\in C_{I}}d\left(x_{i},c_{I}\right)+\frac{1}{\left|C_{J}\right|}\sum_{x_{i}\in C_{J}}d\left(x_{i},c_{J}\right)}{d\left(c_{I},c_{J}\right)}\right),$$

where $\left|C_{I}\right|$ represents the cell number of cluster $C_{I}$, $c_{I}=\frac{1}{\left|C_{I}\right|}\sum_{x_{i}\in C_{I}}x_{i}$ is the centroid of cluster $C_{I}$, and d(⋅,⋅) denotes the Euclidean distance function.

Both SC and DBi are used to evaluate clustering performance. SC ranges in [−1,1], where a value closer to one indicates more comprehensive clustering performance. DBi ranges in [0,+∞), where a value closer to zero indicates more satisfactory clustering performance.

## AUTHOR CONTRIBUTIONS

**Xiang Wang:** Data curation; methodology; formal analysis; visualization; writing‐original draft preparation. **Sansheng Yang:** Data curation; validation; visualization. **Hongwei Li:** Conceptualization; funding acquisition; supervision; writing‐review and editing.

## CONFLICT OF INTEREST STATEMENT

The authors Xiang Wang, Sansheng Yang, and Hongwei Li declare that they have no conflicts of interest.

## ETHICS STATEMENT

This article does not contain any studies with human or animal materials performed by any of the authors.

## Supporting information

Supporting Information S1

## Data Availability

The source codes are available at the GitHub website (EnchantedJoy/scSCC).
