# Supplementary material for: scSCC: A swapped contrastive learning‐based clustering method for single‐cell gene expression data
Source: Quant Biol. 2025 Feb 5;13(2):e85. doi: 10.1002/qub2.85 (PMC12806040; doi:10.1002/qub2.85)
Supplement: Supplementary file 1 — Supporting Information S1 [file QUB2-13-e85-s001.pdf]

Supplementary Materials – "scSCC: A swapped contrastive learning based clustering method for single-cell gene expression data"

Xiang Wang, Sansheng Yang, Hongwei Li

June 21, 2024

Supplementary Figures

Figure S1. Comparison results of SC and DBi.
Figure S2. Comparison results of ARI and NMI of 10 methods.
Figure S3. Time consumption of 5 deep learning-based methods across 10 datasets.
Figure S4. t-SNE visualization of scSCC over ten datasets with the predicted labels.
Figure S5. t-SNE visualization of scSCC over ten datasets with the ground-truth labels.
Figure S6. ARI results of kmeans and scSCC.
Figure S7. NMI results of kmeans and scSCC.
Figure S8. Comparison results of ARI and NMI of different noise ratios.
Figure S9. Cell-cell similarities with different masking probabilities of Trachea dataset.
Figure S10. t-SNE visualization of cell representations of Trachea dataset when $p_m = 0.1$.
Figure S11. t-SNE visualization of cell representations of Trachea dataset when $p_m = 0.9$.
Figure S12. ARI scores under different settings of τ_i and τ_s.

Supplementary Tables

Table S1. ARI scores of eight methods over ten datasets.
Table S2. NMI scores of eight methods over ten datasets.
Table S3. ARI scores of ten methods over ten datasets.
Table S4. NMI scores of ten methods over ten datasets.
Table S5. ARI scores of different selections of the hyperparameter κ.
Table S6. NMI scores of different selections of the hyperparameter κ.
Table S7. A summary of the ten real scRNA-seq datasets.

1. Supplementary Figures

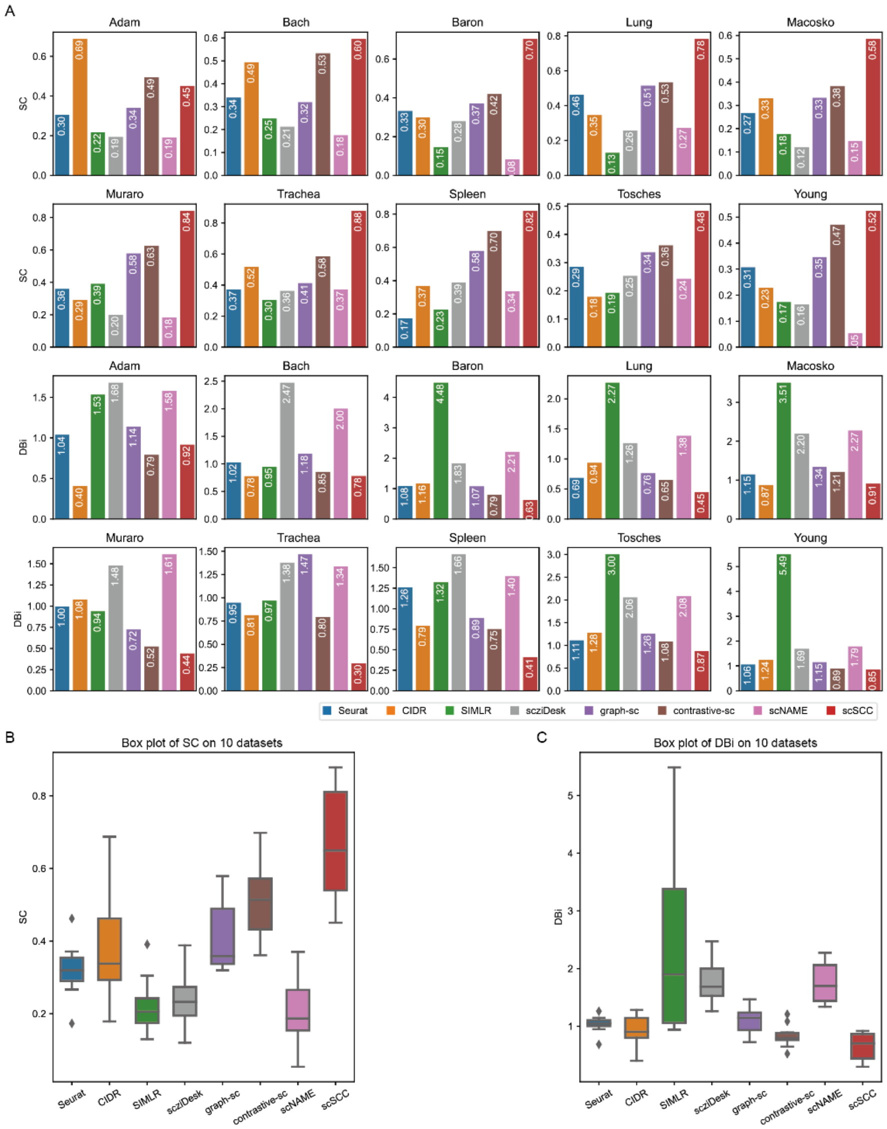

Figure S1: Comparison results of SC and DBi. (A) Bar charts of SC and DBi scores of the eight algorithms on ten datasets. The first two rows are SC scores and the last two rows are DBi scores. (B) Box plots of SC scores of the eight algorithms over ten datasets. (C) Box plots of DBi scores of the eight algorithms over ten datasets. In the box plot, the center line, box limits and whiskers denote the median, upper and lower quartiles, and 1.5× interquartile range, respectively.

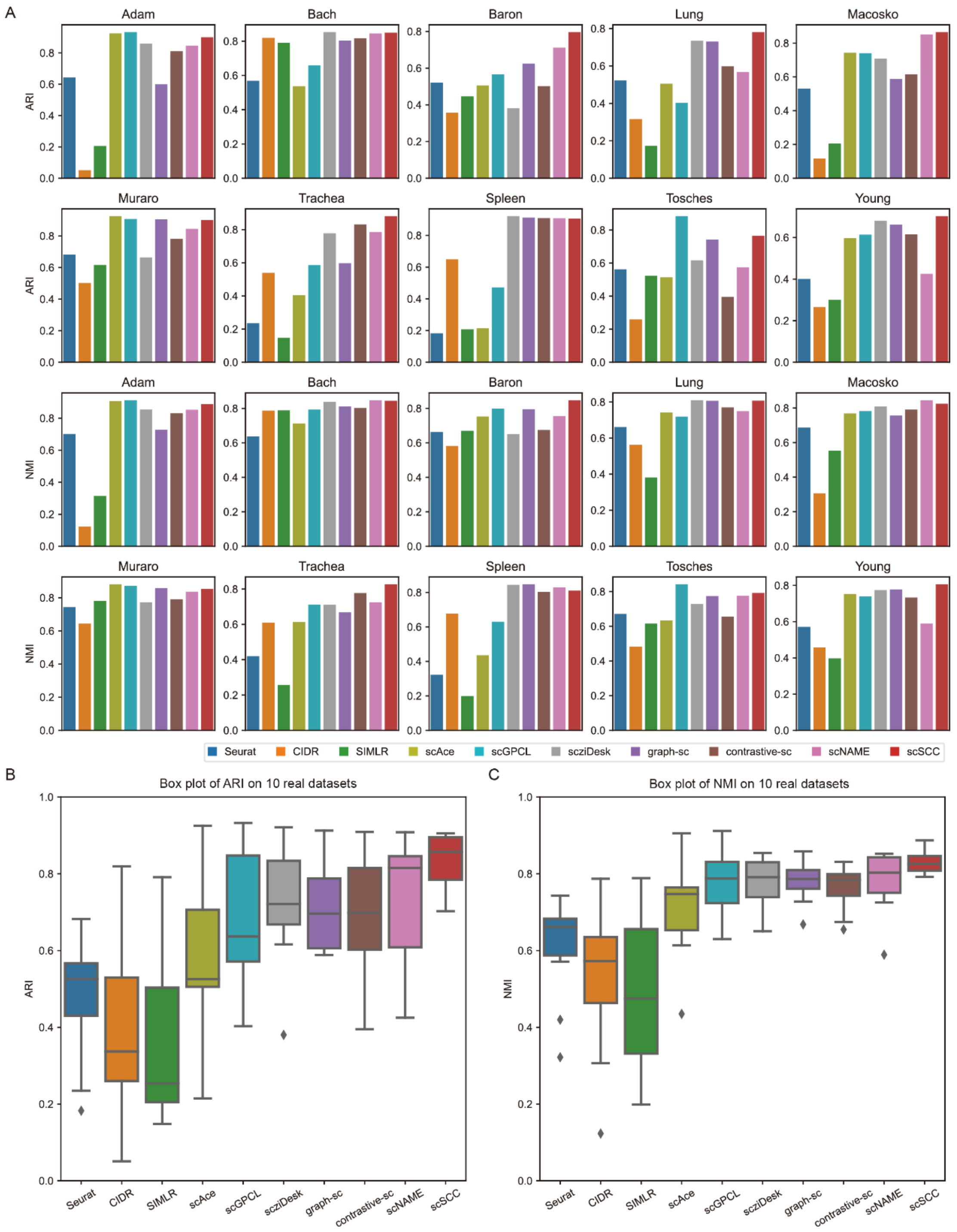

Figure S2: Comparison results of ARI and NMI of 10 methods. (A) Bar charts of ARI and NMI scores of the ten algorithms on ten datasets. The first two rows are ARI scores and the last two rows are NMI scores. (B) Box plots of ARI scores of the ten algorithms over ten datasets. (C) Box plots of NMI scores of the ten algorithms over ten datasets. In the box plot, the center line, box limits and whiskers denote the median, upper and lower quartiles, and 1.5× interquartile range, respectively.

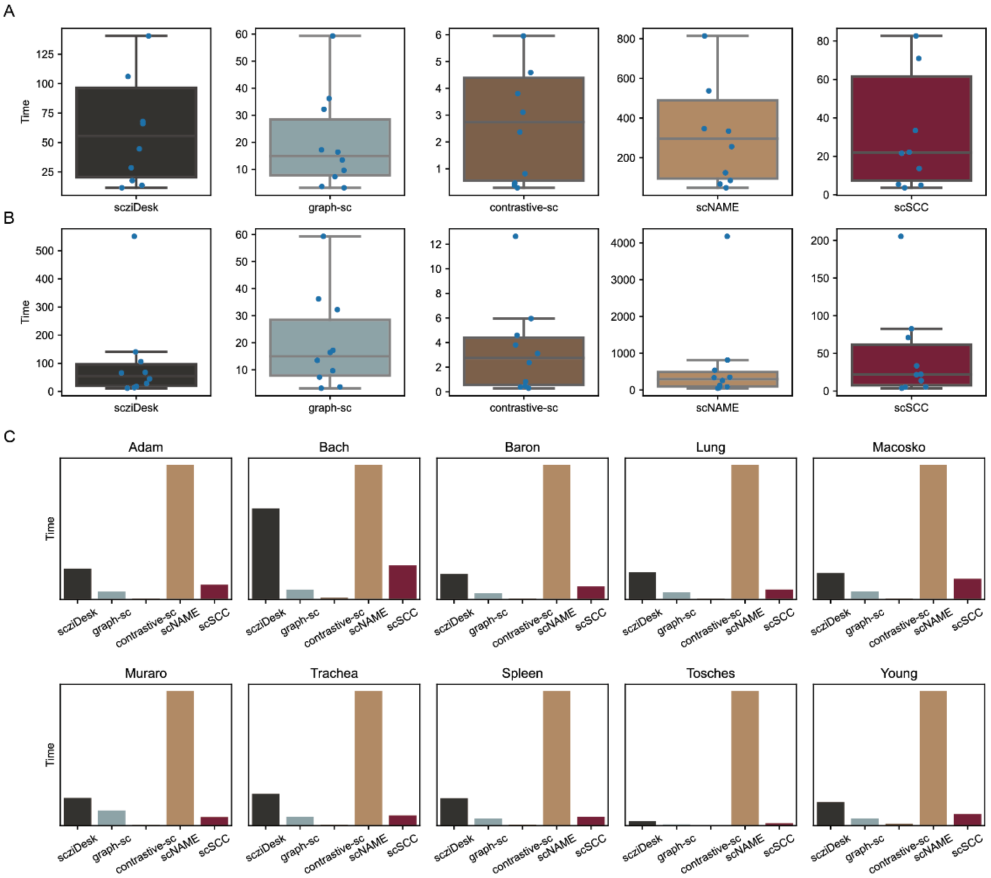

Figure S3: Time consumption of 5 deep learning-based methods across 10 datasets. (A) Box plots of time consumption of the 5 methods across ten datasets after removing outliers. (B) Box plots of time consumption of the 5 methods across ten datasets. (C) Bar plots of time consumption of the 5 methods on each dataset. In the box plot, the center line, box limits and whiskers denote the median, upper and lower quartiles, and 1.5× interquartile range, respectively.

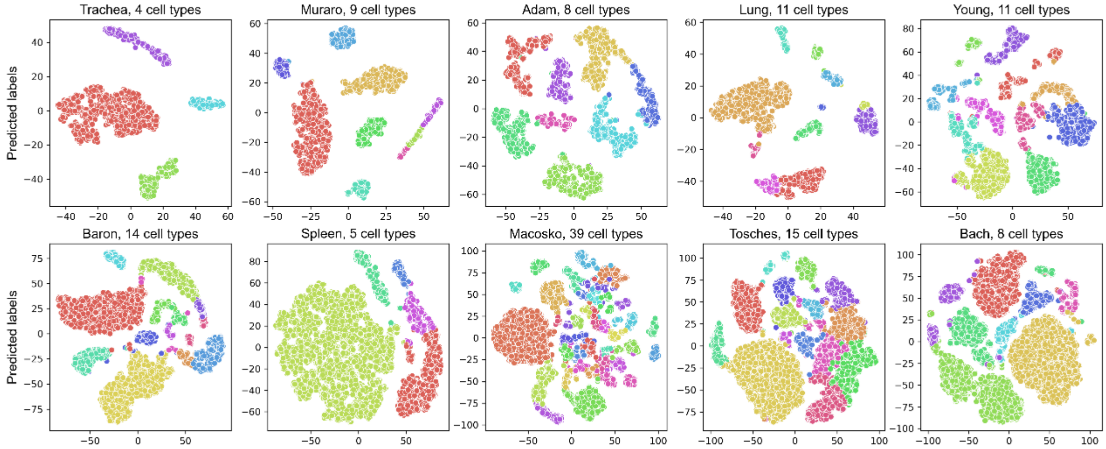

Figure S4: t-SNE visualization of scSCC over ten datasets with the predicted labels. Each color denotes a cell type, and each spot denotes a cell.

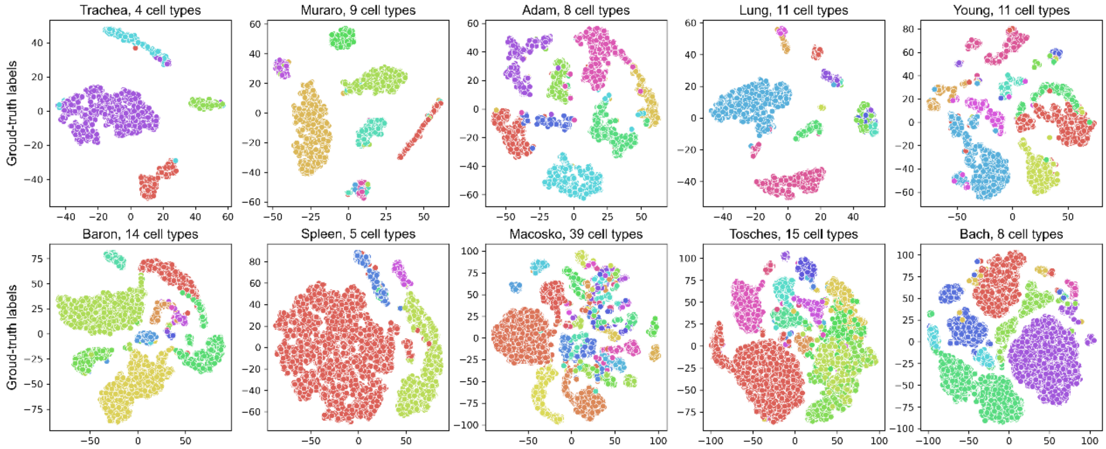

Figure S5: t-SNE visualization of scSCC over ten datasets with the ground-truth labels. Each color denotes a cell type, and each spot denotes a cell.

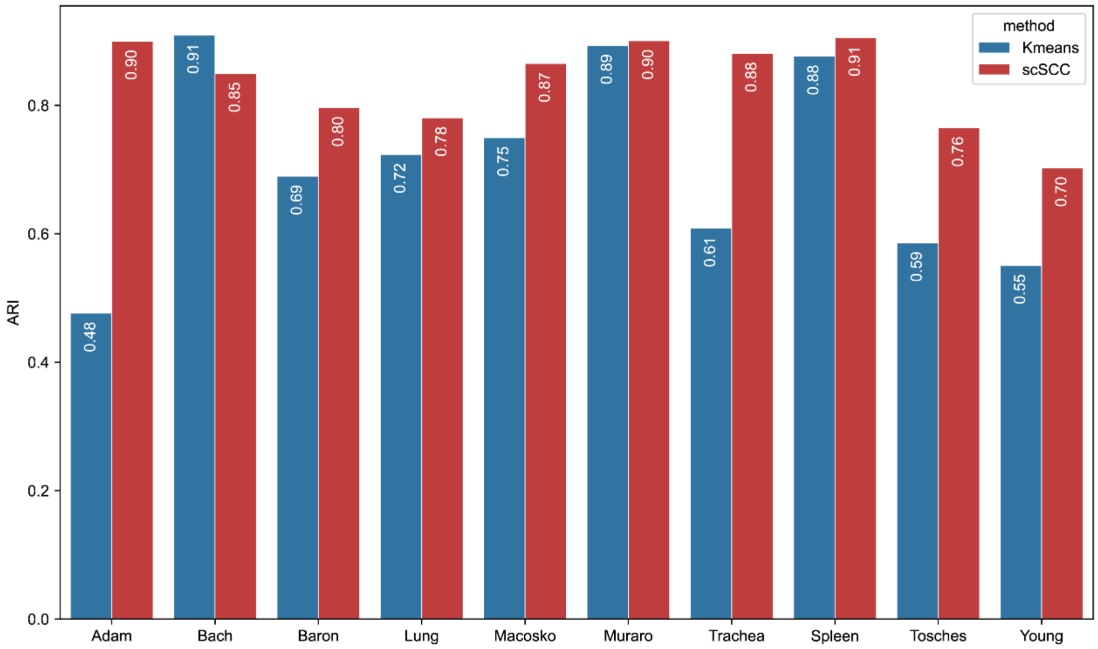

Figure S6: ARI results of kmeans and scSCC.

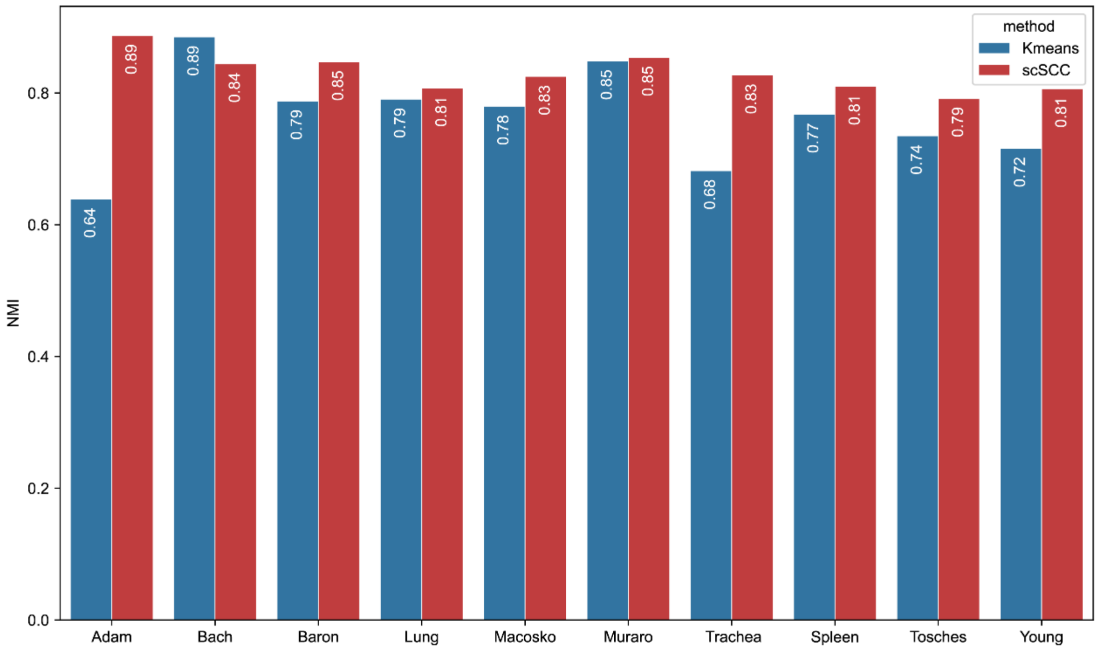

Figure S7: NMI results of kmeans and scSCC.

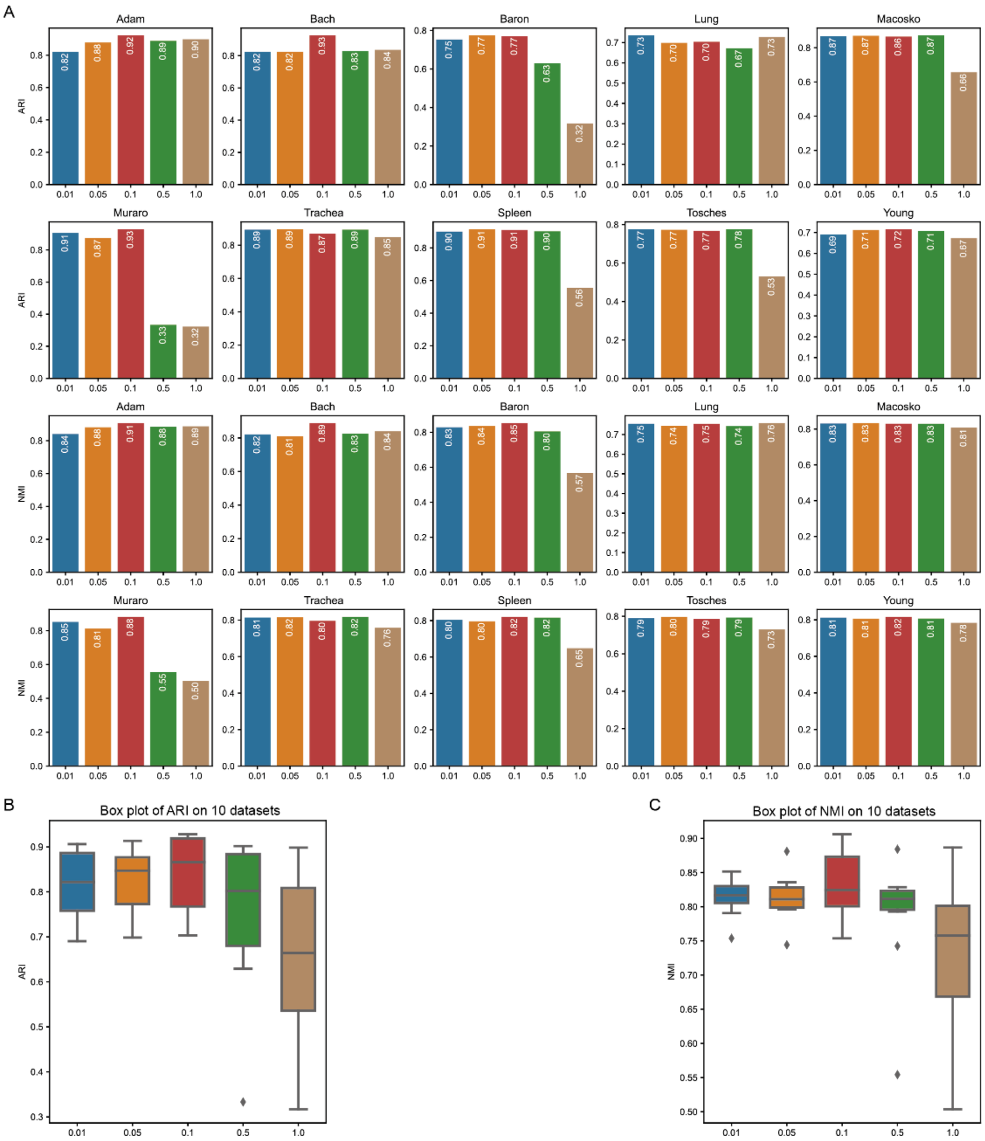

Figure S8: Comparison results of ARI and NMI of different noise ratios. (A) Bar charts of ARI and NMI scores of different noise ratios on ten datasets. The first two rows are ARI scores and the last two rows are NMI scores. (B) Box plots of ARI scores of different noise ratios over ten datasets. (C) Box plots of NMI scores of different noise ratios over ten datasets. In the box plot, the center line, box limits and whiskers denote the median, upper and lower quartiles, and 1.5× interquartile range, respectively.

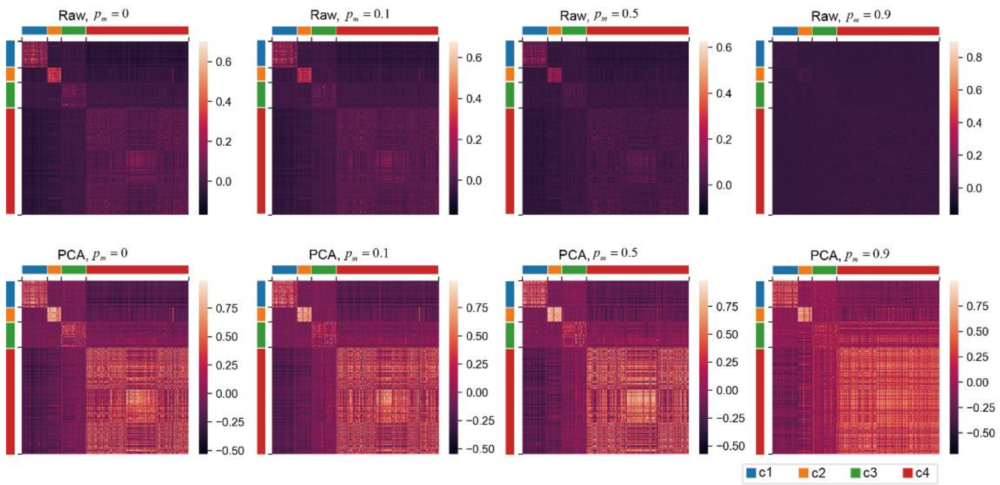

Figure S9: Cell-cell similarities with different masking probabilities of Trachea dataset. Heatmaps in the first row show the cell-cell similarities calculated from the preprocessed expression vectors under different masking probabilities. Heatmaps in the second row how the cell-cell similarities calculated from the PCA expression vectors under different masking probabilities. The similarities are represented by cosine similarity.

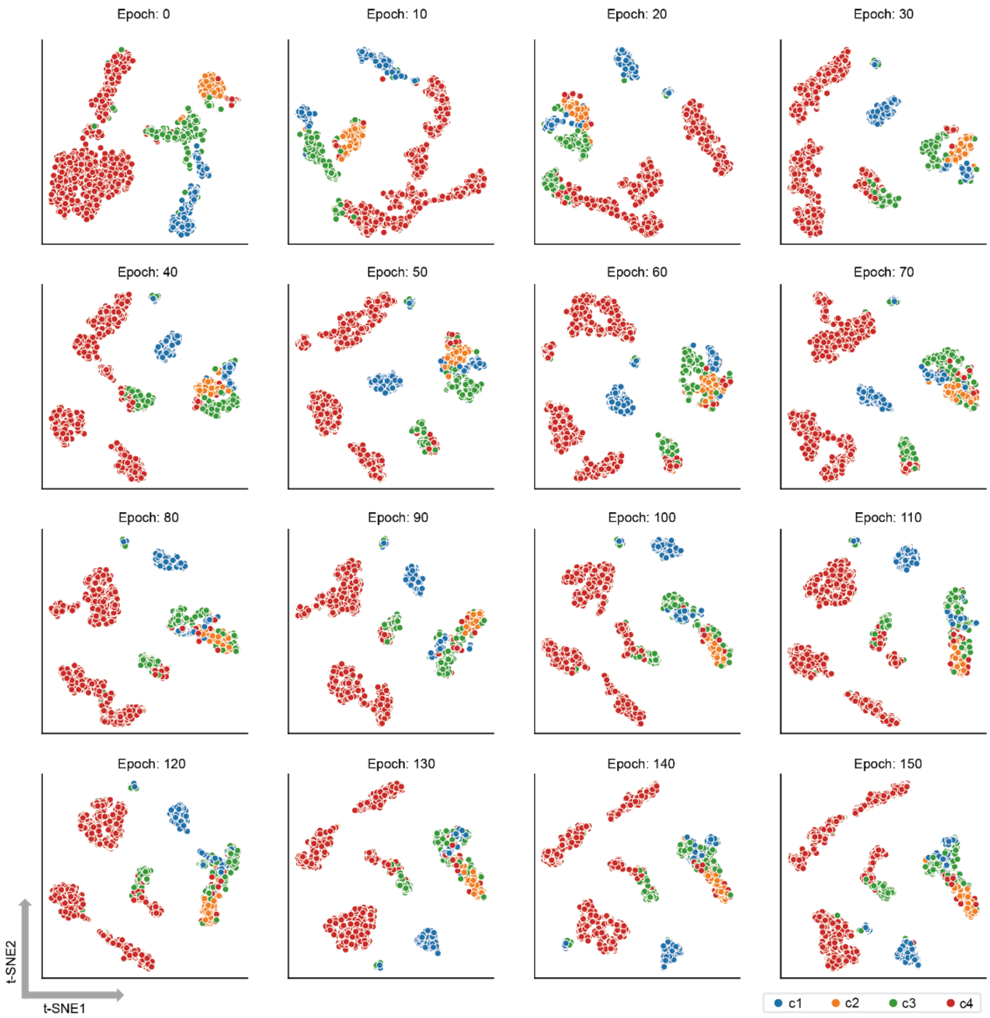

Figure S10: t-SNE visualization of cell representations of Trachea dataset when $p_m = 0.1$. Each spot represents a cell and each color represents a cell type.

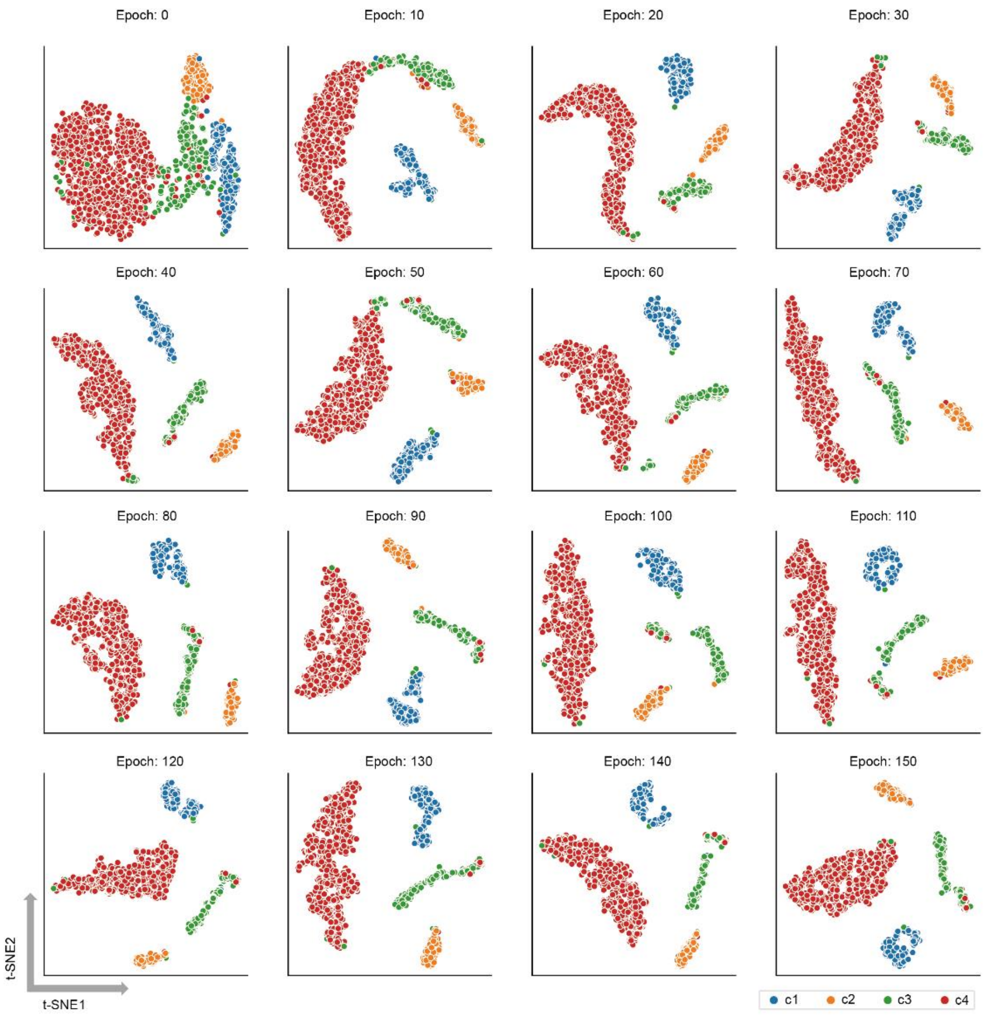

Figure S11: t-SNE visualization of cell representations of Trachea dataset when $p_m = 0.9$. Each spot represents a cell and each color represents a cell type.

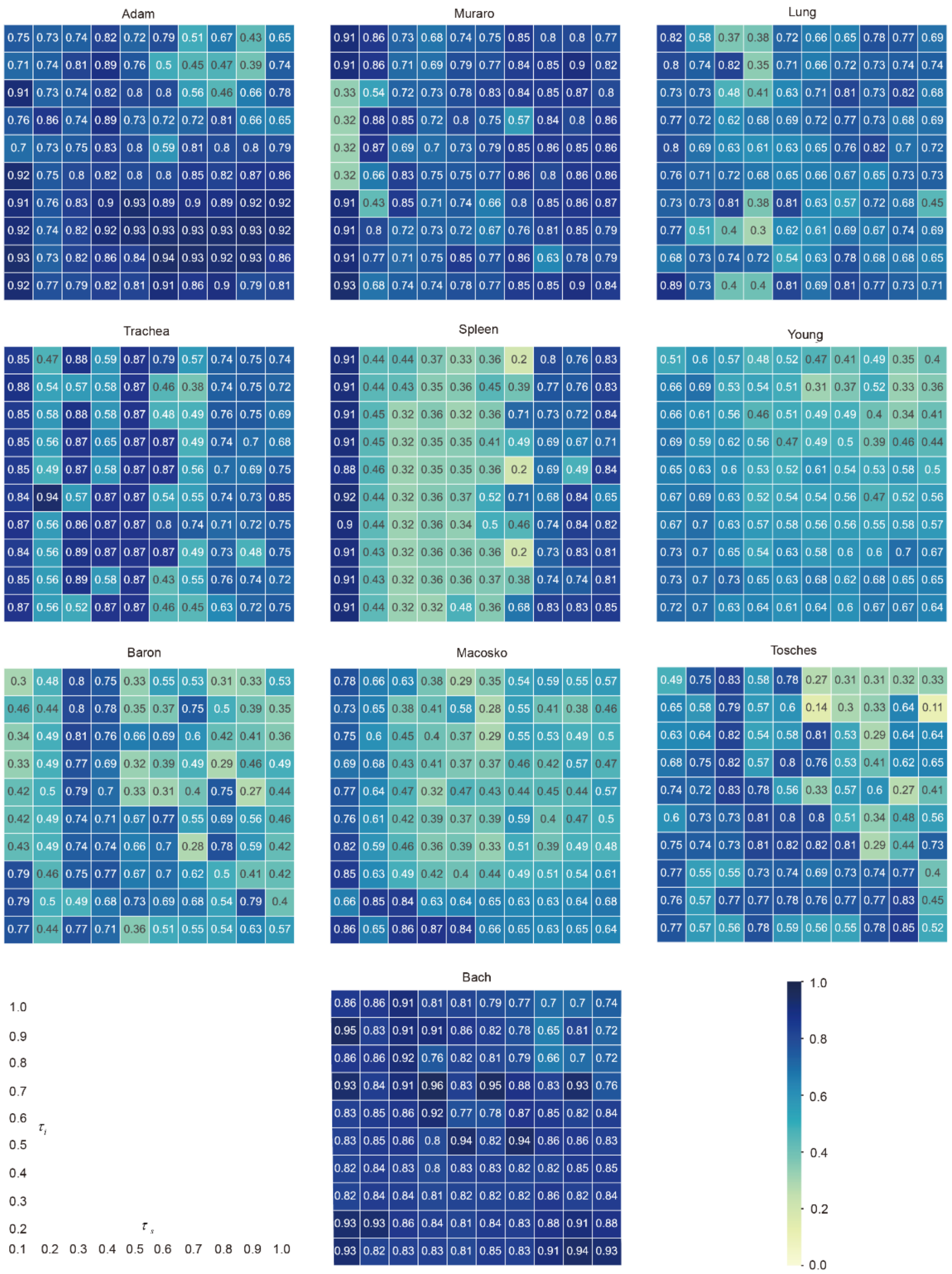

Figure S12: ARI scores under different settings of τ_i and τ_s. Both τ_i and τ_s increase from 0.1 to 1 in intervals of 0.1. The default setting of scSCC was located in the bottom-left corner of each dataset.

2. Supplementary Tables

Table S1: ARI scores of eight methods over ten datasets. Bold scores represent the best, and scores with * represents the second best.

Dataset	Seurat	CIDR	SIMLR	scziDesk	graph-sc	contrastive-sc	scNAME	scSCC
Muraro	0.6823	0.5019	0.616	0.6639	**0.9042**	0.7818	0.8446	0.9006*
Adam	0.6432	0.051	0.205	0.8593*	0.5996	0.8109	0.8461	**0.9**
Young	0.4004	0.2656	0.3001	0.6804*	0.6622	0.6148	0.425	**0.7025**
Baron	0.5206	0.3574	0.446	0.3803	0.6245	0.5022	0.7116*	**0.7962**
Macosko	0.5294	0.1168	0.2057	0.7088	0.5884	0.6156	0.8512*	**0.8651**
Tosches	0.5621	0.2586	0.5226	0.616	0.7422*	0.3952	0.5746	**0.7649**
Bach	0.5685	0.8194	0.7912	**0.8528**	0.8029	0.8164	0.8453	0.8496*
Trachea	0.2346	0.5387	0.1481	0.7779	0.597	0.8317*	0.7855	**0.881**
Lung	0.5225	0.3162	0.1726	0.7342*	0.73	0.5981	0.5684	**0.7807**
Spleen	0.1826	0.6484	0.2077	**0.9212**	0.9126*	0.9088	0.9084	0.9055

Table S2: NMI scores of eight methods over ten datasets. Bold scores represent the best, and scores with * represents the second best.

Dataset	Seurat	CIDR	SIMLR	scziDesk	graph-sc	contrastive-sc	scNAME	scSCC
Muraro	0.743	0.644	0.7803	0.772	**0.8586**	0.7907	0.8367	0.854*
Adam	0.701	0.1231	0.3146	0.854*	0.7278	0.831	0.8521	**0.8872**
Young	0.5709	0.4571	0.3971	0.7741	0.7777*	0.7336	0.5892	**0.8064**
Baron	0.6626	0.582	0.6698	0.6502	0.7952*	0.6746	0.7553	**0.8472**
Macosko	0.6871	0.3064	0.5527	0.8082	0.7568	0.791	0.8452*	**0.8252**
Tosches	0.6713	0.4825	0.6151	0.7279	0.774	0.6549	0.7756*	**0.7919**
Bach	0.6363	0.7872	0.7887	0.8372	0.8111	0.802	**0.8474**	0.8444*
Trachea	0.4198	0.6103	0.2559	0.7108	0.6682	0.7772*	0.7253	**0.8271**
Lung	0.6612	0.5628	0.3812	**0.8099**	0.807	0.7696	0.7489	0.8077*
Spleen	0.322	0.6767	0.1989	0.8438*	**0.8472**	0.8035	0.8297	0.8101

Table S3: ARI scores of ten methods over ten datasets. Bold scores represent the best, and scores with * represents the second best.

Dataset	Seurat	CIDR	SIMLR	scAce	scGPCL	scziDesk	graph-sc	contrastive-sc	scNAME	scSCC
Adam	0.6432	0.0510	0.2050	0.9249*	**0.9325**	0.8593	0.5996	0.8109	0.8461	0.9000
Bach	0.5685	0.8194	0.7912	0.5369	0.6590	**0.8528**	0.8029	0.8164	0.8453	0.8496*
Baron	0.5206	0.3574	0.4460	0.5054	0.5666	0.3803	0.6245	0.5022	0.7116*	**0.7962**
Lung	0.5225	0.3162	0.1726	0.5052	0.4029	0.7342*	0.7300	0.5981	0.5684	**0.7807**
Macosko	0.5294	0.1168	0.2057	0.7430	0.7399	0.7088	0.5884	0.6156	0.8512*	**0.8651**
Muraro	0.6823	0.5019	0.6160	**0.9252**	0.9074*	0.6639	0.9042	0.7818	0.8446	0.9006
Trachea	0.2346	0.5387	0.1481	0.4049	0.5862	0.7779	0.5970	0.8317*	0.7855	**0.8810**
Spleen	0.1826	0.6484	0.2077	0.2149	0.4723	**0.9212**	0.9126*	0.9088	0.9084	0.9055
Tosches	0.5621	0.2586	0.5226	0.5137	**0.8836**	0.6160	0.7422	0.3952	0.5746	0.7649*
Young	0.4004	0.2656	0.3001	0.5963	0.6139	0.6804*	0.6622	0.6148	0.4250	**0.7025**

Table S4: NMI scores of ten methods over ten datasets. Bold scores represent the best, and scores with * represents the second best.

Dataset	Seurat	CIDR	SIMLR	scAce	scGPCL	scziDesk	graph-sc	contrastive-sc	scNAME	scSCC
Adam	0.7010	0.1231	0.3146	0.9057*	**0.9120**	0.8540	0.7278	0.8310	0.8521	0.8872
Bach	0.6363	0.7872	0.7887	0.7130	0.7931	0.8372	0.8111	0.8020	**0.8474**	0.8444*
Baron	0.6626	0.5820	0.6698	0.7531	0.7982*	0.6502	0.7952	0.6746	0.7553	**0.8472**
Lung	0.6612	0.5628	0.3812	0.7420	0.7190	**0.8099**	0.8070	0.7696	0.7489	0.8077*
Macosko	0.6871	0.3064	0.5527	0.7686	0.7824	0.8082	0.7568	0.7910	**0.8452**	0.8252*
Muraro	0.7430	0.6440	0.7803	**0.8815**	0.8729*	0.7720	0.8586	0.7907	0.8367	0.8540
Trachea	0.4198	0.6103	0.2559	0.6136	0.7127	0.7108	0.6682	0.7772*	0.7253	**0.8271**
Spleen	0.3220	0.6767	0.1989	0.4349	0.6299	0.8438*	**0.8472**	0.8035	0.8297	0.8101
Tosches	0.6713	0.4825	0.6151	0.6332	**0.8421**	0.7279	0.7740	0.6549	0.7756	0.7919*
Young	0.5709	0.4571	0.3971	0.7522	0.7392	0.7741	0.7777*	0.7336	0.5892	**0.8064**

Table S5: ARI scores of different selections of the hyperparameter κ, The bold value is the highest score for specific dataset, and the value with $*$ is the score under the default setting.

Dataset	$\kappa = 0.01$	$\kappa = 0.1$	$\kappa = 1$	$\kappa = 5$	$\kappa = 10$
Adam	0.75	0.84	**0.9***	0.9	0.89
Bach	0.88	0.85	0.85*	**0.89**	0.92
Baron	**0.8***	0.56	0.51	0.51	0.5
Lung	**0.78***	0.64	0.47	0.47	0.47
Macosko	0.81	0.85	**0.87***	0.67	0.65
Muraro	**0.9***	0.88	0.69	0.69	0.7
Trachea	**0.88***	0.54	0.53	0.52	0.53
Spleen	**0.91***	0.45	0.49	0.33	0.33
Tosches	0.48	0.74	0.76*	0.77	**0.78**
Young	0.52	0.66	0.7*	0.69	**0.73**

Table S6: NMI scores of different selections of the hyperparameter κ, The bold value is the highest score for specific dataset, and the value with $*$ is the score under the default setting.

Dataset	$\kappa = 0.01$	$\kappa = 0.1$	$\kappa = 1$	$\kappa = 5$	$\kappa = 10$
Adam	0.82	0.86	**0.89***	0.89	0.88
Bach	0.87	0.85	0.84*	0.88	**0.89**
Baron	**0.85***	0.78	0.77	0.77	0.77
Lung	**0.81***	0.78	0.72	0.73	0.73
Macosko	0.73	0.8	**0.83***	0.82	0.81
Muraro	**0.85***	0.85	0.8	0.8	0.8
Trachea	**0.83***	0.66	0.64	0.62	0.63
Spleen	**0.81***	0.62	0.63	0.56	0.57
Tosches	0.68	0.76	**0.79***	0.79	0.79
Young	0.7	0.78	0.81*	0.81	**0.82**

Table S7: A summary of the ten real scRNA-seq datasets. For the Baron dataset, the cell type annotations were obtained based on expression of unique transcripts and with reference to literature, after an iterative hierarchical clustering analysis. For the Muraro dataset, the cell type annotations were obtained based on top differentially expressed genes and the classical marker genes , after StemID clustering. For the Macosko dataset, the cell type annotations were obtained based on differential expression testing, after density-based clustering. For the Young dataset, the cell type annotations were determined based on marker genes curated from literature, after graph-based clustering. For the Adam, Tosches and Bach datasets, the cell type annotations were obtained based on known marker genes, after graph-based clustering. For the Trachea, Spleen and Lung datasets, the cell type annotations were obtained based on known marker genes after graph-based clustering, and then debiased by the FACS sorting.

Dataset	Size(cells × genes)	Cell types	Source
Muraro	2122 × 19046	9	GEO accession number GSE85241
Adam	3660 × 23797	8	GEO accession number GSE87544
Young	5685 × 33658	11	EGA accession number EGAS00001002171
Baron	8569 × 20125	14	GEO accession number GSM2230758
Macosko	49300 × 24658	39	GEO accession number GSE63473
Tosches	18664 × 23500	15	NCBI accession number PRJNA408230
Bach	23184 × 19965	8	GEO accession number GSE106273
Trachea	1350 × 23341	4	GEO accession number GSE109774
Lung	1676 × 23341	11	GEO accession number GSE109774
Spleen	9552 × 23341	5	GEO accession number GSE109774

The filtered Macosko dataset is located at https://figshare.com/articles/dataset/scDCC_data/21563517.
